# Supplementary material for: Brain-Wide Transgene Expression in Mice by Systemic Injection of Genetically Engineered Exosomes: CAP-Exosomes
Source: Pharmaceuticals (Basel). 2024 Feb 20;17(3):270. doi: 10.3390/ph17030270 (PMC10976217; doi:10.3390/ph17030270)
Supplement: Supplementary file 1 [file pharmaceuticals-17-00270-s001.zip › pharmaceuticals-2759057-supplementary.pdf]

Supplemental Figure S1

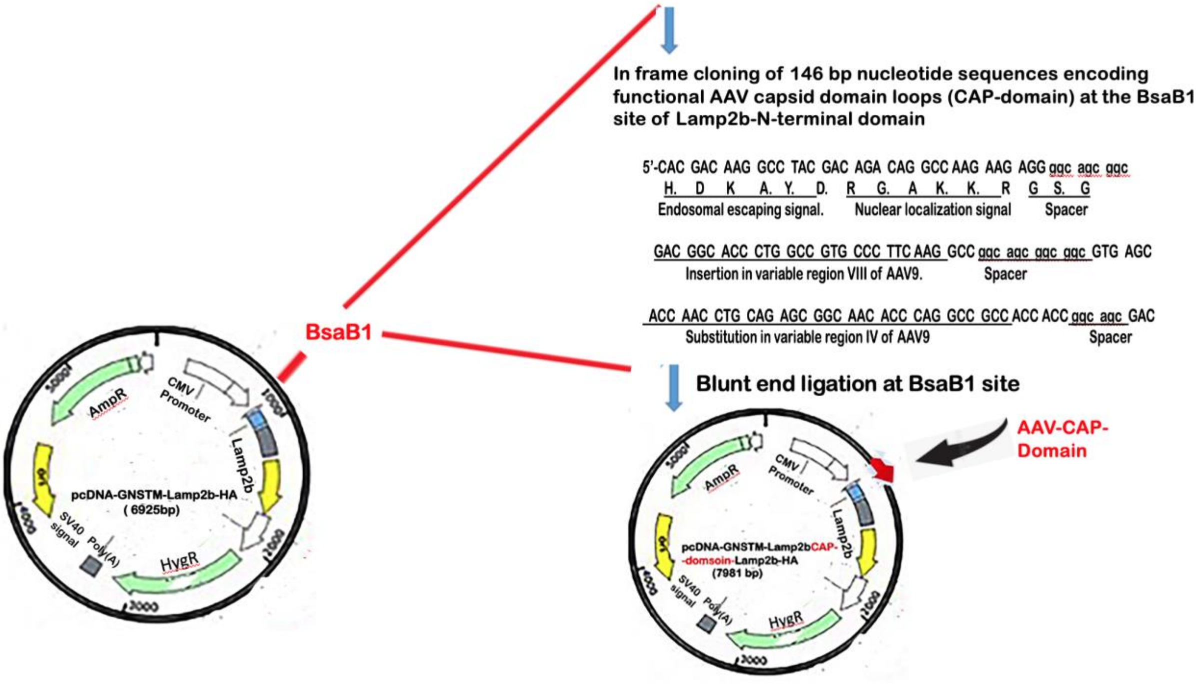

Supplementary Figure S1: Graphical presentation of cloning strategy for AAV-CAP domain in pcDNA GNSTM-3-Flag-10-lamp2b-HA expression plasmid vector. (Addgene, USA, Plasmid no.71293)
